# Nutrition management for patients with head and neck cancer during peri-radiotherapy: A systematic review and quality appraisal of clinical practice guidelines using the AGREE II instrument

**DOI:** 10.3389/fonc.2022.974059

**Published:** 2022-11-29

**Authors:** Jing Zhao, Yajing Kan, Xueting Wu, Shuang Yang, Guozhou Wang, Yuting Bao, Jing Li

**Affiliations:** ^1^Key Laboratory of Cancer Prevention and Therapy, National Clinical Research Center for Cancer, Tianjin’s Clinical Research Center for Cancer, Tianjin Medical University Cancer Institute and Hospital, Tianjin, China; ^2^Graduate School of Tianjin Medical University, Tianjin, China

**Keywords:** head and neck cancer, radiotherapy, nutrition management, nutrients, clinical practice guidelines

## Abstract

**Objective:**

To evaluate the quality of clinical practice guidelines (CPGs) for nutrition management of patients with head and neck cancer (HNC) during peri-radiotherapy, as well as to summarize the nutrition recommendations fitting the subject.

**Methods:**

CPGs published in English, Chinese and German were identified from databases, guideline networks, and websites of nutritional associations from the databases’ inception to March 8, 2022. Three independent appraisers used the Appraisal of Guidelines for Research and Evaluation II (AGREE II) Instrument to assess the quality of CPGs. The intraclass correlation coefficient (ICC) was used to calculate appraiser agreement.

**Results:**

769 records were identified. After removing duplicates, 470 articles were screened. 12 CPGs were identified with nutrition-specific recommendations. 67% of CPGs were rated as high quality, and 33% as low quality. Recommendations were categorized into nutritional risk screening, nutrition assessment, nutrition counseling, nutrition interventions, nutrition intake, swallowing function management, weight management, exercise, multidisciplinary team, post-discharge care, nutrients, and pharmacologic interventions.

**Conclusion:**

We found discrepant recommendations in existing CPGs, including nutrition screening, nutrition assessment, nutrition intake, and nutrients. We also reported the absence of essential parts of CPGs, including the views of its target users, the statement of external review, the method to formulate the recommendations, strategies to improve uptake, and resource implications of applying the CPGs. CPGs with low quality should be improved in future updates based on currently available guideline development tools. Specialized CPGs on nutrition management for HNC patients during peri-radiotherapy should be developed.

**Systematic review registration:**

https://www.crd.york.ac.uk/PROSPERO/index.php, identifier CRD42022320322.

## Introduction

Head and neck cancers (HNCs) arise from major anatomical sites: the oral cavity, paranasal sinuses, sinonasal cavity, pharynx, salivary glands, and larynx (1, 2). HNC was the seventh most common cancer worldwide in 2020 (930,000 new cases and 470,000 deaths) (3, 4). Radiotherapy (RT) has an integral role for HNC patients. In the primary treatment setting of HNC, RT can provide similar results to surgical treatment for specific early-stage HNCs. For most locally advanced HNCs, RT is a fundamental component of comprehensive therapy (5).

Malnutrition is a common complication of cancer and may reduce therapeutic effects. About two million cancer patients worldwide die yearly due to severe malnutrition, accounting for 30% of cancer patients (6). It is well known that HNC patients are frequently malnourished before starting treatment (7). RT may cause side effects, such as xerostomia, mucositis, nausea and vomiting, alteration or loss of taste, and consequent worsening malnutrition (8). A study by Abu et al. (9) reported that the mean weight loss of HNC patients was 7.4% during RT treatment and 2.1% post-treatment. Furthermore, deterioration of the nutritional status leads to an increase in RT-related toxicity, may decrease the responses to RT, and prolong treatment duration (8).

Healthcare professionals are responsible for providing patients with safe and high-quality treatment and care based on the best existing evidence (10). Guidelines promote high-quality cancer care. Clinical practice guidelines (CPGs) are formulated according to specific procedures, and high-quality CPGs serve as evidence-based resources to provide healthcare professionals with better decisions in clinical circumstances (11, 12). Many CPGs related to cancer nutrition management have been published, whereas those may vary dramatically in quality. Moreover, inconsistency across CPGs may dilute high-quality recommendations and increase confusion in clinical practice (13, 14). The Appraisal of Guidelines for Research and Evaluation II (AGREE II) was developed to evaluate the quality of reporting and the practice guideline development (15). The AGREE II Instrument has been widely used and validated since it was updated in 2010. Ng et al. (16) assessed the quality of HNC guidelines for complementary and alternative medicine recommendations and found that quality varied among the guidelines. Zhou et al. (17) evaluated the quality of CPGs of the nutritional risk for cancer patients, the majority of CPGs were rated as ‘strongly recommended’ or ‘recommended with modifications’.

Regarding our search, there has been no systematic review of CPGs on nutrition management for HNC population during peri-radiotherapy. This review appraised the quality of the relevant CPGs using the AGREE II Instrument and extracted nutrition recommendations for the target population to provide information for establishing nutritional care practice standards and developing or updating CPGs.

## Materials and methods

### Design

This review sought to identify nutrition management CPGs for HNC population using standard methods (18) and the Preferred Reporting Items for Systematic Reviews and Meta-Analyses (PRISMA) criteria (19, 20). A protocol was registered with PROSPERO (CRD42022320322, May 7, 2022). Eligible CPGs were assessed with the AGREE II Instrument, which includes six domains: scope and purpose, stakeholder involvement, rigor of development, clarity of presentation, applicability, and editorial independence (21). Each item in six domains and the overall assessment item in the AGREE II Instrument are rated on a 7-point scale (1-strongly disagree to 7-strongly agree). The user’s manual states that each domain score is independent and should not be added to a single quality score (21). As no specific patient data was involved, ethics approval from the institutional review board was not applicable.

### Search strategies

A comprehensive search was conducted from databases’ inception to March 8, 2022 by two independent reviewers (YK and XW) in the following data sources: (a) eight electronic databases, including PubMed, Web of Science, Excerpta Medica dataBASE (EMBASE), Cochrane Library, China National Knowledge Infrastructure (CNKI), Wanfang Database China, Biomedical Literature Service System (SinoMed) and Weipu Information Chinese Periodical Service Platform (VIP); (b) eleven guideline databases, including National institute for Health and Care Excellence (NICE), Scottish Intercollegiate Guidelines Network (SIGN), National Comprehensive Cancer Network (NCCN), National Guideline Clearinghouse (NGC), Guideline International Network (GIN), New Zealand Guidelines Group (NZGG), Queensland Clinical Guidelines (QCG), Australian Government National Health and Medical Research Council (NHMRC), Association of the Scientific Medical Societies in Germany (AWMF), Medlive and MedSci; and (c) three professional nutrition society websites, including European Society for Clinical Nutrition and Metabolism (ESPEN), America Society for Parenteral and Enteral Nutrition (ASPEN) and Chinese Society of Parenteral and Enteral Nutrition (CSPEN).

We combined subject terms, for instance, MeSH terms, with entry terms for searches in four databases: PubMed, Cochrane Library, EMBASE, and SinoMed. In the remaining databases, free text terms (limited to title, abstract, or keywords) were used. Keywords included ‘nutrition*/diet*/malnutrition/sarcopenia/anorexia/cachexia’, ‘head and neck cancer*/neoplasm*/carcinoma*/tumor*’, ‘radiotherapy/radiation therapy/chemoradiotherapy’ and ‘guideline*/CPG*/clinical practice guideline*’.

All articles available as full-text versions were considered, and if the full-text or supplemental material was not available, an inquiry was sent to the author/guideline panel. The search strategy for each electronic database has been included in **Supplementary Material 1**. The literature search was repeated on May 1, 2022, and yielded no new CPGs eligible for this study.

### Selection criteria

Inclusion criteria included (a) the eligible CPGs published in English, Chinese or German; (b) CPGs covering recommendations for nutrition management for HNC patients; (c) healthcare-professional-used CPGs; and (d) CPGs in the latest versions.

Exclusion criteria included (a) quick reference CPGs or abridged versions of the CPGs published alongside the CPGs; (b) publications in the form of statements, protocols, abstracts, conference proceedings, letters, editorials commentaries, or CPG interpretations; (c) consensus-based CPGs; (d) translated versions of original CPGs included before in this review; and (e) CPGs specific to one country or region.

### Literature screening and data extraction

All records were imported to EndNote X9, and duplicates were removed automatically and by manual checking. The titles and abstracts of the outputs were screened independently by two reviewers (YK and XW) to select the potential CPGs. Then, the full text of each potential CPG was further assessed for eligibility. The reviewers also screened the reference lists of eligible CPGs to identify further relevant CPGs. All eligible CPGs were finally included after discussions between the reviewers.

Two reviewers (YK and XW) extracted the following information: author, year of publication or update, country or region, major development organization (academic institutions, government agencies, disease-specific foundations, or professional associations or societies), CPG topic, target group, grading system and whether there were nutritional experts in the CPG development panel.

### Guideline quality assessment

All documents related to the CPGs (full CPG document, appendices, supplementary material, and journal publications) were collected for analysis. Three appraisers (YK, XW, and GW) assessed CPGs independently with the AGREE II Instrument. Before the assessment, a meeting was held to discuss the appraisal criteria according to the AGREE II manual and training tools. When the difference in score of a single item > 2 existed among the appraisers, discussions were conducted to agree until the difference achieved 2 or less.

Domain scores were calculated by summing up all the scores of included individual items and by scaling the total as a percentage of the maximum possible score for that domain. The scaled domain score (ranges from 0% to 100%) was calculated *via* the following formula: (obtained score – minimum possible score)/(maximum possible score − minimum possible score) × 100%. Domains were then compared based on these percentages. Higher scaled domain scores related to a higher quality of the guidelines.

The AGREE II Instrument does not offer the cut-off scores to determine quality of domains or whole of the guideline, so the decision on the quality level of CPGs was made by the appraisers’ evaluation of the scaled domain scores of the six domains. To determine the overall quality, the appraisers rated CPGs as high quality when five or more domains scored above 60%; average quality represented three to four domains scored above 60%; and two or fewer domains scored above 60% were considered low quality, according to previous literature (14, 22). In this study, the average score of 23 items indicates whether it is supposed to be recommended for clinical use. The average score above 6 suggests ‘yes’, with recommending the CPG for use; the score ranging from 4 to 6 presents ‘yes, with modifications’; and the score below 4 means ‘no’ for recommendation.

The Excel 2019 software was used to manage the data of the CPGs. The inter-rater agreement was presented by the intraclass correlation coefficient (ICC) *via* IBM SPSS 26.0 software. An ICC > 0.75 indicates satisfactory consistency, 0.5 ≤ ICC ≤ 0.75 is considered as generally acceptable consistency, and ICC < 0.5 suggests unsatisfactory consistency.

### Recommendation extraction

Only the CPGs rated as high-quality or average-quality with the AGREE II Instrument were submitted to the recommendation extraction process by two reviewers (YK and XW). The chapters regarding nutrition management in CPGs were first identified and then screened for specific recommendations. The nutrition management spectrum included nutritional risk screening, nutrition assessment, nutrition interventions, nutrition intake, nutrition status, nutrients, dysphagia, enteral nutrition, parenteral nutrition, nutritional supplementary, feeding tube, oral nutrition, exercise, weight management, multidisciplinary team, follow-up, post-discharge care and so on.

## Results

### Literature search

**Figure 1:**
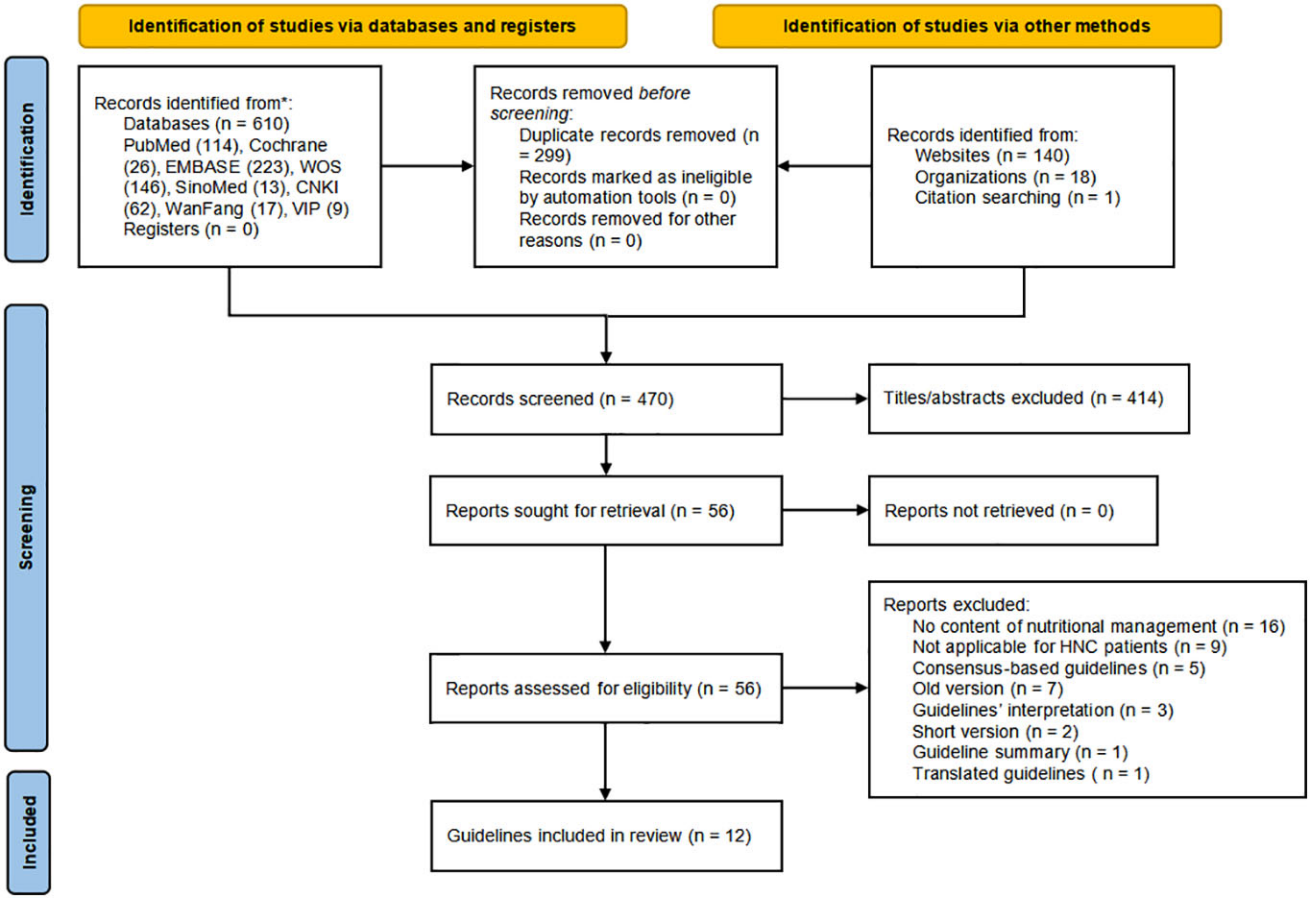
Flow chart of the literature retrieval and selection process.

The PRISMA flowchart presents the literature retrieval and selection process in **Figure 1**. **Supplemental Material 1** provides the database search findings which yielded 769 records, including 610 from electronic databases and 159 from relevant websites. After manual and software automatic duplicates removal, 470 articles were identified. The remaining articles were scrutinized in the titles and abstracts for covering nutrition recommendations for HNC patients, 414 articles were further excluded. Then 56 guidelines were sought for retrieval and assessed for eligibility, of which 12 CPGs (23–34) were finally included in the assessment.

### Characteristics of included CPGs

Eligible CPGs were published or updated from 2015 to 2022 (50% were in the last five years) in the Europe (n = 1), UK (n = 1), US (n = 5), Spain (n = 1), Australia (n = 1), China (n = 2) and Germany (n = 1). Four CPGs (24, 29, 31, 34) were developed for HNC patients, and eight CPGs (23, 25–28, 30, 32, 33) for cancer patients partly dedicated to nutrition management for HNC patients. The nutritional funding sources were found in four CPGs (23, 26, 32, 34). All CPGs included nutrition experts as part of the development panel except one (24). **Table 1** shows the characteristics across the included CPGs.

**Table 1:** The characteristics of included CPGs.

Guideline	First author Year	Country/Region	Development institution(major)	Guideline topic	Target group	Grading system	Nutritional experts in the CPG development panel
ESPEN practical guideline: Clinical Nutrition in cancer (23)	Maurizio Muscaritoli et al. (2021)	Europe	European Society for Clinical Nutrition and Metabolism, ESPEN	Offer optimal nutritional care to cancer patients	Cancer patients	Studies: GRADE Recomm: Strong, Weak	Yes
Head and Neck Cancers, Version 2.2022, NCCN Clinical Practice Guidelines in Oncology (24)	David G. Pfister et al. (2022)	USA	National Comprehensive Cancer Network, NCCN	Diagnosis, treatment, and management for HNC patients	HNC patients	Study: NCCN Categories of Evidence and Consensus Recomm: All Recommendations are considered appropriate in this guideline	Not mentioned
Survivorship, Version 1.2022, NCCN Clinical Practice Guidelines in Oncology (25)	Tara Sanft et al. (2022)	USA	National Comprehensive Cancer Network, NCCN	Screening, evaluation, and treatment for cancer survivors.	Cancer survivors	Study: NCCN Categories of Evidence and Consensus Recomm: All Recommendations are considered appropriate in this guideline	Yes
Oncology Evidence-Based Nutrition Practice Guideline for Adults (26)	Kyle L. Thompson et al. (2017)	USA	Academy of Nutrition and Dietetics, AND	Oncology nutrition for the care of adult patients with cancer	Adult cancer patients	Studies: EAL Recomm: Strong, Fair, Weak, Consensus, Insufficient	Yes
Management of Cancer Cachexia: ASCO Guideline (27)	Eric J Roeland et al. (2020)	USA	American Society of Clinical Oncology, ASCO	Clinical management of cancer cachexia in adult patients with advanced cancer	Adult patients with advanced cancer	Studies: GLIDES Recomm: Strong, Moderate, Weak	Yes
Guidelines for clinical diagnosis and treatment of cancer cachexia (28)	Jiuwei Cui et al. (2020)	China	China Anti-Cancer Association, CACA	Screening, diagnosis, evaluation, and treatment of cancer cachexia	Cancer patients	Studies: Delphi Recomm: A-E	Yes
American Cancer Society Head and Neck Cancer Survivorship Care Guideline (29)	Ezra E W Cohen et al. (2016)	USA	American Cancer Society, ACS	HNC survivors care	Adult head and neck cancer survivors (post-treatment)	Studies: LOE Recomm: Not mentioned	Yes
Guidelines on nutritional support in patients with tumor (30)	Guohao Wu et al. (2017)	China	Chinese Society for Parenteral and Enteral Nutrition, CSPEN	Nutritional support for cancer patients	Cancer patients	Studies: GRADE Recomm: Strong, Weak	Yes
Nutritional management in head and neck cancer: United Kingdom National Multidisciplinary Guidelines (31)	Talwar B et al. (2016)	UK	UK Multidisciplinary Guidelines	Nutritional management in HNC patients	HNC patients	No criteria, solely based on the included studies	Yes
S3-Guideline of the German Society for Nutritional Medicine (DGEM) in Cooperation with the DGHO, the ASORS and the AKE, Clinical Nutrition in Oncology (32)	J. Arends et al. (2015)	Germany	German Society for Nutritional Medicine, DGEM	Diagnosis and multi-modal treatment of nutritional and metabolic problems in cancer patients	Cancer patients	Studies: Not mentioned, but there was evidence level: Ia, Ib, IIa, IIb, III Recomm: A, B, C, KKP	Yes
SEOM clinical guidelines on nutrition in cancer patients (2018) (33)	R. de las Peñas et al. (2018)	Spain	Spanish Society of Medical Oncology, SEOM	Nutritional intervention in cancer	Cancer patients	Studies: USPSTF Recomm: A-E	Yes
Evidence-based practice guidelines for the nutritional management of adult patients with head and neck cancer (34)	Merran Findlay et al. (2016, the time of latest update of guideline’s content)	Australia	Clinical Oncology Society of Australia, COSA	Nutritional management for adult HNC patients	Adult HNC patients	Studies: NHMRC Recomm: A-D	Yes

GRADE, Grading of Recommendations, Assessment, Development and Evaluations; EAL, Evidence Analysis Library; GLIDES, Guidelines Into Decision Support; LOE, Levels of Evidence; USPSTF, US Agency for Healthcare Research and Quality Service Grading System; NHMRC, National Health and Medical Research Council; Recomm, Recommendations.

### Quality assessment

The results appraised with the AGREE II Instrument are presented in **Supplementary Material 2**. The average score, scaled domain scores, and the ICC are shown in **Table 2**. Results of average score showed that four CPGs (23, 27, 29, 34) scored above 6, with ‘yes’ for recommendation; four CPGs (24–26, 32) scored 4 ~ 6, with ‘yes, with modification’; and the remaining CPGs (28, 30, 31, 33) scored 3 ~ 4, with ‘no’ for recommendation. Based on the scaled scores of six domains, eight CPGs were rated as high quality (23–27, 29, 32, 34), and four as low quality (28, 30, 31, 33). The lowest domain score was for ‘stakeholder involvement’ (domain 2), with a median score of 56.48% (ranging from 25.93% to 96.30%). The ‘clarity of presentation’ (domain 4) got the highest scores and the minor variability with a median score of 98.15% (ranging from 74.07% to 100%). The lowest mean score (2.75) for an individual item was ‘The guideline has been externally reviewed by experts before its publication’ (Item 13). The ICC ranged from 0.766 to 0.990, indicating satisfactory consistency.

**Table 2:** Domains and total scores for included CPGs with the AGREE II Instrument.

Guideline	Scope and purpose	Stakeholder involvement	Rigor of development	Clarity of presentation	Applicability	Editorial independence	Average score	Quality level	ICC (95%CI)
ESPEN (23)	81.48%	87.04%	75.00%	100.00%	80.56%	100.00%	6.03	High	0.916 (0.841 to 0.961)
NCCN (24)	85.19%	42.59%	60.42%	96.30%	73.61%	100.00%	5.30	High	0.952 (0.907 to 0.978)
NCCN (25)	81.48%	68.52%	72.22%	96.30%	72.22%	100.00%	5.71	High	0.930 (0.866 to 0.967)
AND (26)	87.04%	55.56%	78.47%	100.00%	69.44%	100.00%	5.78	High	0.935 (0.875 to 0.970)
ASCO (27)	100.00%	96.30%	93.75%	100.00%	93.06%	100.00%	6.77	High	0.766 (0.532 to 0.893)
CACA (28)	77.78%	42.59%	52.78%	74.07%	22.22%	0.00%	3.86	Low	0.974 (0.949 to 0.988)
ACS (29)	100.00%	92.59%	91.67%	100.00%	100.00%	100.00%	6.77	High	0.980 (0.961 to 0.991)
CSPEN (30)	79.63%	25.93%	49.31%	100.00%	25.00%	0.00%	3.90	Low	0.990 (0.979 to 0.995)
UK Multidisciplinary Guidelines (31)	72.22%	53.70%	13.19%	94.44%	33.33%	0.00%	3.35	Low	0.983 (0.967 to 0.992)
DGEM (32)	81.48%	57.41%	72.22%	96.30%	61.11%	97.22%	5.49	High	0.920 (0.848 to 0.962)
SEMO (33)	62.96%	27.78%	27.78%	96.30%	38.89%	47.22%	3.70	Low	0.953 (0.909 to 0.978)
COSA (34)	100.00%	94.44%	95.83%	100.00%	94.44%	100.00%	6.81	High	0.825 (0.686 to 0.915)
All guidelines (minimum-maximum, median)	62.96%-100.0%, 81.48%	25.93%-96.30%, 56.49%	13.19%-95.83%, 72.22%	74.07%-100%, 98.15%	22.22% - 100%, 70.83%	0.00% - 100%, 100%	3.35-6.81, 5.60	–	–

ESPEN, European Society for Clinical Nutrition and Metabolism; NCCN, National Comprehensive Cancer Network; AND, Academy of Nutrition and Dietetics; ASCO, American Society of Clinical Oncology; CACA, China Anti-Cancer Association; ACS, American Cancer Society; CSPEN, Chinese Society for Parenteral and Enteral Nutrition; DGEM, German Society for Nutritional Medicine; SEOM, Spanish Society of Medical Oncology; COSA, Clinical Oncology Society of Australia.

#### Scope and purpose

This domain involves the primary purpose, specific clinical issues, and target population of the guidelines (21). All the CPGs scored > 60%, and three CPGs scored 100% (27, 29, 34). All appraised CPGs made statements with reasonable clarity. The SEOM guideline (33) got the lowest score of 62.96%, for the descriptions of purpose, clinical questions, and intended audience, were not clear and concise enough without labeled sections or chapters.

#### Stakeholder involvement

This domain is concerned with the extent to which the guideline was developed by the appropriate stakeholders and whether the recommendations represent the views of its target users (21). Five CPGs (23, 25, 27, 29, 34) scored > 60%, one CPGs (30) received the lowest score (25.93%). Most CPGs mentioned some of the following information: members of the development group, institutional affiliation, geographical location, and subject discipline, but only two (27, 34) described members’ roles in the guideline development group. Five CPGs (23, 27, 29, 32, 34) provided involvement details of target population, of which the DGEM guideline’s (32) statement was found in the general standards of AWMF-S3 guidelines development on the website. The target users were poorly defined in three CPGs (30, 32, 33).

#### Rigor of development

This domain pertains to the process used to gather and synthesize the evidence, the methods to formulate the recommendations, and update them (21). Eight CPGs (23–27, 29, 32, 34) scored > 60%, and one (31) received the lowest score (13.19%). Two CPGs (31, 33) did not use systematic methods to search for evidence, and four (24, 30, 31, 33) did not mention the criteria for selecting evidence. The strengths and limitations of the body of evidence were clearly described for the majority of CPGs, but not for one (31). Eight CPGs (23–25, 27, 28, 30, 32, 34) elaborated on the method of formulating the recommendations, but two (31, 33) omitted it. For most CPGs, the health benefits, side effects, and risks were primarily considered in formulating the recommendations except one (31). All CPGs met the goal of an explicit link between the recommendations and the supporting evidence. Three CPGs (27, 29, 34) had been externally reviewed by experts before publication, whereas others had poor/no description. Eight CPGs (23–27, 29, 32, 34) included a procedure for updating, while four (28, 30, 31, 33) did not. The ESPEN guideline was developed based on the ESPEN-specific framework, which is available online (23). The method of DGEM guideline (32) was described in detail in the guideline report (including search strategy and evidence table et al.) on the website of the Association of Scientific Medical Societies (AWMF). The ASCO Guideline Methodology Manual provided the update process and a summary of literature search results in the **Data Supplement** (27). The ACS guideline (29) and COSA guideline (34) published a comprehensive list of evidence available online, and the COSA guideline (34) also provided search strategy on the website.

#### Clarity and presentation

This domain mainly concerns the guideline’s language, structure, and format (21). All the CPGs approached or came up to (23–27, 29–34) scores of 100% except one (28), which scored 74.07% because the key recommendations were not easily identifiable.

#### Applicability

This domain focuses on the potential facilitators and barriers to application, auditing criteria, measures to improve transmitting power, and resource implications of applying the guideline (21). Eight CPGs (23–27, 29, 32, 34) scored > 60%, two (28, 30) scored < 30% that only mentioned the monitoring/auditing criteria. Only three CPGs (27, 29, 34) provided complete description of applicability, by contrast, two (28, 30) had no mention of facilitators and barriers to application, four (28, 30, 31, 33) barely presented the tools and/or advice for applying the recommendations, and three (28, 30, 31) scarcely considered potential resource implications of applying the recommendations.

#### Editorial independence

This domain is to evaluate whether the formulation of recommendations was unduly biased by competing interests (21). Conflict of interest is the most common source of bias in guideline development (35). Eight CPGs (23–27, 29, 32, 34) approached or came up to a score of 100%. The SEMO guideline (33) scored 47.22% that reported competing interests without statements of funding bodies’ influence for recommendations, and three (28, 30, 31) received a score of 0.00% for no disclosure of editorial independence. Five CPGs (24–26, 32, 34) provided an explicit statement of interest disclosure and editorial independence on their official website.

### Extraction of recommendations

Classified recommendations for nutrition management were integrated into specific sections in **Table 3**, which included nutritional risk screening (24, 26, 32, 34), nutrition assessment (23, 24, 26, 32, 34), nutrition counseling (23, 24, 32, 34), nutrition interventions (23, 24, 32, 34), nutrition intake (23, 27, 32, 34), swallowing function management (23, 24, 29, 34), weight management (25, 34), exercise (23, 25, 27, 32), multidisciplinary team (24, 26, 27, 34) and post-discharge care (24, 25, 29, 34). The nutrients (23, 25–27, 32, 34) and pharmacologic interventions (23, 27, 32, 34) were summarized separately in **Table 4** in view of the volume and complexity.

**Table 3:** Recommendations for nutrition management.

**Nutritional risk screening**	Patients receiving adjuvant radiotherapy are at high risk of malnutrition and supposed to be closely monitored for nutritional impact symptoms such as dysphagia, time and effort while eating, and long-standing side effects of treatment, e.g., xerostomia and dysgeusia (34).
	All HNC patients should be evaluated for nutrition risks using a validated screening tool at diagnosis (24, 32, 34) (e.g., Malnutrition Screening Tool (26, 34), Malnutrition Universal Screening Tool (26, 32), Nutritional risk screening 2002 (32)), and repeated at intervals during each phase of treatment (e.g., radiotherapy/chemotherapy, surgery, and post-treatment) (34).
**Nutrition assessment**	Patients who are identified as being at risk of malnutrition by nutrition screening should receive further nutrition assessment for weight change, food intake, nutrition-related symptoms, body and muscle mass, physical performance, and systemic inflammation (23, 32).
	Nutrition assessment should be undertaken before cancer diagnosis and repeated according to the stability of the patient’s clinical situation (23).
	The nutrition assessment should be conducted via validated tools (e.g., Patient-Generated Subjective Global Assessment, Subjective Global Assessment) from pre to post treatment (24, 26, 34).
	Nutrition status should be monitored closely in patients who have: 1) significant weight loss (5% weight loss over the prior 1 month, or 10% weight loss more than 6 months); and/or 2) dysphagia due to tumor lesions or pain with the initiation of treatment (24).
**Nutrition counseling**	HNC patients should counsel a dietitian about nutrition status before radiotherapy (24) and repeat it weekly during the treatment (34).
	Cancer patients involved in malnutrition, especially who are receiving anti-tumor treatment, should take nutrition counseling (23).
	To increase oral food intake, nutrition consultation should be provided to cancer patients (32).
**Nutrition interventions**	Tube feeding should be taken to minimize weight loss and improve nutrition status for patients with intolerant oral intake (34). Enteral nutrition (EN) if oral intake is still insufficient, and parenteral nutrition (PN) if EN remains inadequate or available (32).
	For obstructive HNC tumors or severe mucositis, it is recommended to implement enteral feeding with nasogastric tubes (23, 32).
	Clinicians should discuss with patients about the way of tube placement for individualized nutrition care (34).
	If oral food intake is seriously insufficient for a long time, the nutrient intake should be slowly increased within a few days for preventing refeeding syndrome (23, 32).
	It is not recommended to place preventive PEG or NG tubes if patients are in good condition without severe dysphagia, significant airway obstruction, or serious weight loss before treatment (24).
	Prophylactic feeding tube placement is recommended for patients with 1) severe weight loss prior to treatment; 2) significant complications due to lack of food intake, difficulty in swallowing, or weak tolerance of dehydration; 3) ongoing dysphagia or dehydration, anorexia, or inadequate eating/drinking caused by pain; 4) slight aspiration but with compromised cardiopulmonary function or age > 60 years; 5) severe aspiration; 6) long-term swallowing disorders or large area of high-dose radiation to the adjacent connective tissues and mucosa; or 7) hypopharyngeal or T4 tumors and receive concurrent chemoradiotherapy at the same time (24, 34).
	Patients who do not need placement of prophylactic PEG or NG tubes prior to treatment should be monitored weekly for treatment-related side effects, weight changes, and caloric intake during treatment (24).
**Nutrition intake**	The supply of vitamins and minerals should be equal to the recommended daily intake, and the high-dose of micronutrients is discouraged for use (23).
	The energy intake should be above 125 kJ/kg/day (30 kcal/kg/day) and the protein intake should be at least 1.0~2.0 g/kg/day and, if possible up to 1.5 g/kg/day, for patients who receive radiotherapy or chemoradiotherapy (23, 27, 32, 34). Nutrition intake and weight should be monitored regularly to determine whether energy requirements are met or not (34).
**Swallowing function management**	Clinicians should screen and manage dysphagia and encourage patients to maintain swallowing function during EN (23).
	A baseline assessment of swallowing and speech is recommended for patients involved in dysfunction for swallowing and/or speech (24).
	Alterations of swallowing function may occur for a prolonged period after therapy (especially after radiotherapy) and should be monitored and managed during the lifetime of the patients (24).
	Primary care clinicians should: a) refer HNC survivors with unexplained weight loss, pneumonia, dysphagia, and/or postprandial cough to speech-language pathologists for swallowing function assessment to manage possible aspiration and dysphagia; b) recognize suspected psychosocial obstacles of swallowing recovery and refer HNC patients to the clinician if obstacles are present; c) refer HNC survivors with potential stricture to a speech-language pathologist for videofluoroscopy; d) refer HNC survivors with the symptom of stricture to dilation surgery or gastroenterologist (29).
	It is recommended that the patient maintain nutrition status with safe swallowing before the tube removal (34).
**Weight management**	Survivors should be evaluated for body composition, metabolic health, and primary care settings, and achieve metabolic health, normal body mass index (BMI) and adjusted interventions as appropriate, for the protein and energy requirements remain high after treatment (25, 34).
	Weight management mainly includes physical activity, behavior modification, and caloric management (25).
	It is uncertain to recommend weight loss supplements for cancer survivors (25).
**Exercise**	Exercise therapy should be guided by a trained person and combined with nutritional therapy to increase muscle mass (32).
	Cancer patients should maintain or increase their physical activity level to support metabolic patterns, physical function, and muscle mass (23).
	Primary care clinicians should encourage HNC survivors to take regular physical activity and specifically: a) avoid inactivity and come back to normal daily activities as early as possible; b) achieve 75 min of vigorous or up to 150 min of moderate aerobic exercise each week (25).
**Multidisciplinary team**	Multidisciplinary team or rehabilitation team should involve a language/swallowing therapist and a registered dietitian for cancer patients who are receiving CT or RT or require tube feeding post treatment (24, 26, 34).
	Primary care clinicians should refer cancer patients who lose body weight, appetite and/or taste to a registered dietitian for dietary counseling and assistance (27, 29).
**Post-discharge care**	Nutrition interventions should be provided for at least 3 months post treatment (34).
	HNC survivors should avoid extreme temperature liquids, acidic or citric liquids, sugary soft drinks or sugar-containing chewing gum, spicy or abrasive foods, alcohol or consume alcohol sparingly, and tobacco, and take sufficient whole grains, dietary fiber, fruits, and vegetables and low saturated fats (25, 29).
	Cancer survivors should obtain nutrients from food sources rather than dietary supplements which are not suitable for regular use (25).
	Dietitian should assess cancer survivors’ dietary patterns of daily intake, the timing of meals, portion size, frequency of eating out and snacking habits (25).
	Cancer survivors are recommended to take: 1) fat: plant sources and fatty fishes; 2) carbohydrates: legumes, whole grains, vegetables, and fruits; 3) protein: poultry, fish, nuts, legumes, and low-fat dairy foods; 4) soy foods: moderate consumption (≤ 3 servings/day) due to its uncertain effects in cancer control (25).
	The patient should receive follow-up by the dietitian post treatment: 1) at least a fortnight review over 6 weeks; 2) as required for up to 6 months; 3) as long as they require management of weight loss, tube feeding, or chronic toxicities; 4) continue until a stable nutrition baseline is achieved (24, 34).

**Table 4:** Recommendations for pharmacologic interventions and nutrients.

CPGs Items	ESPEN (23)	NCCN (25)	AND (26)	ASCO (27)	DGEM (32)	COSA (34)
Corticosteroids	To increase the appetite, corticosteroids may be used for a restricted period (1-3 weeks), and side effects (e.g., muscle wasting, insulin resistance, infections) should be monitored.	–	–	3-4 mg dexamethasone equivalent dose may be used to improve appetite.	Corticosteroids limited to a few weeks may be used to relieve loss of appetite, and possible adverse reactions should be considered.	–
Progestins	To increase the appetite, progestins may be used, and side effects (e.g., thromboembolism) should be monitored.	–	–	A short-term trial of a progesterone analog may be used for patients experiencing loss of appetite and/or body weight.	Progesterone may be used to relieve the loss of appetite, and possible adverse reactions should be considered.	–
Cannabinoids	Uncertain recommendations on cannabinoids to improve taste disorders or anorexia in cancer patients.	–	–	Based on the current evidence, cannabinoids should not be used for cancer cachexia management.	Cannabinoids may be used to improve the appetite of patients with tumor cachexia and taste disorders.	–
Androgens	Uncertain recommendation on androgenic steroids to increase muscle mass.	–	–	Uncertain recommendation on androgens for tumor cachexia management.	Uncertain recommendation on androgenic steroids to increase muscle mass.	–
Thalidomide	-	–	–	Uncertain recommendation on thalidomide for tumor cachexia management.	–	–
Olanzapine or mirtazapine	–	–	–	Uncertain recommendation on olanzapine for tumor cachexia management.	–	–
Nonsteroidal Antiinflammatory Drugs (NSAIDs)	Uncertain recommendation on NSAIDs to improve body weight in weight-losing cancer patients.	–	–	Uncertain recommendation on NSAIDs for tumor cachexia management.	NSAIDs may be used to improve body weight and performance among ward cancer patients.	–
Insulin	–	–	–	–	Insulin combined with other nutritional measures may be used to improve body reserve of patients with tumor cachexia.	–
Anamorelin	–		–	Anamorelin may improve cancer cachexia, but it has not been approved for use.	–	–
Antioxidants	–		–	–	Uncertain recommendation on large doses of antioxidants during chemotherapy.	Antioxidants should not be used during chemotherapy or radiotherapy due to possible tumor protection and survival reducing.
Probiotics	–	–	–	–	Uncertain recommendation on probiotics to reduce radiation-induced diarrhea.	–
Omega-3 fatty acids/N-3 fatty acids (including eicosapentaenoic acid (EPA))	Long-chain N-3 fatty acids or fish oil may be used to stabilize or improve appetite, food intake, lean body mass, and body weight.	–	Dietary supplements containing EPA may be used.	Uncertain recommendation on endorsing omega-3 fatty acids to improve cancer cachexia outcomes.	EPA (1.5-2.5 g/d) may be used to improve systemic inflammatory markers, appetite, food intake, body weight, and quality of life.	–
Glutamine (Gln)	Uncertain recommendation on glutamine to prevent radiation-induced toxic side effects or improve clinical outcomes.	–	Uncertain recommendation on parenteral glutamine to prevent or treat oral mucositis.	–	Glutamine supplementation should not be used to treat malnutrition or cachexia in cancer patients.	–
Hydroxyl methyl butyrate (Leucine metabolite)	–	–	–	–	Hydroxyl methyl butyrate combined with glutamine and arginine may be used to maintain muscle mass in cancer patients.	–
Vitamin E	Vitamins and minerals supplied in amounts approximately equal to the recommended daily allowance may be used, but high-dose micronutrients in the absence of specific deficiencies should not be used.	For most survivors, except in instances of documented deficiencies, inadequate diet, or comorbid indications (e.g., osteoporosis, ophthalmologic disorders, cirrhosis), supplements should not be used.	–	Uncertain recommendation on endorsing vitamins and minerals to improve cancer cachexia outcomes.	–	Vitamin E, at high doses of 400IU/d, should not be used.
Beta carotene			–		–	Beta carotene (30mg/d) may be used carefully to reduce side effects.
Vitamin A			–		–	Vitamin A, at high doses of 200000 IU/week, should not be used.
Zinc			–		–	Zinc (25mg tds) may be used carefully due to potential interactions to chemotherapy and radiotherapy.
Selenium			–		–	Uncertain recommendation on taking selenium of 200ug/d daily during treatment to improve immune function.

Color coding: Light green, ‘should be used’; Light blue, ‘may be used’; Yellow, ‘should not be used’; Orange, ‘uncertain recommendation’.

ESPEN, European Society for Clinical Nutrition and Metabolism; NCCN, National Comprehensive Cancer Network; AND, Academy of Nutrition and Dietetics; ASCO, American Society of Clinical Oncology; DGEM, German Society for Nutritional Medicine; COSA, Clinical Oncology Society of Australia.

## Discussion

HNC patients are at high risk of malnutrition during peri-radiotherapy (36). Clinicians have various obstacles to managing the nutrition status of patients better, such as using clinical judgment when facing ambiguous CPGs (37) or considering the applicability of the international CPGs in the local medical environment. Clinicians need guidance to aid them in making the present decision. Furthermore, clinicians were given quite different recommendations across even highly scoring CPGs sometimes. In that case, several strategies may be adopted, including to search for systematic reviews on quality assessment of related CPGs, which would provide the reference of the included CPGs’ quality level, to select up-to-date and authoritative CPGs if possible, or to use area-specific CPGs, although the latter may compromise the fairness of evidence citation (38). In this review, we also found that high-quality CPGs were mainly produced in developed countries and advanced research centers. It is worth considering whether these CPGs by developed countries apply to developing countries or regions or how to implement them properly in developing countries. Motivating developing countries to develop high-quality CPGs may be another area worth improving. We recommend developing multi-language versions of high-quality CPGs, and an English version must be available to facilitate communication.

To our knowledge, this is the first systematic review of CPGs on nutrition management for HNC population during peri-radiotherapy. Twelve CPGs were included and the recommendations for nutrition management were synthesized. Considering low domain scores with the AGREE II Instrument may be a risk factor for increasing the chance of making incorrect clinical decisions, only recommendations from CPGs of high and average quality were extracted and integrated while those from low quality CPGs were not synthesized, which would probably improve the quality of evidence derived from this study. Similar to other findings from previous studies focusing on CPG assessment of cancer nutrition or management of HNC (16, 17, 38–40), a common finding in this study is the included CPGs’ high heterogeneity of quality. In addition, some of the domains of CPGs, assessed by the AGREE II Instrument, such as ‘Applicability’, ‘Stakeholder involvement’, ‘Editorial independence’ and ‘Rigor of development’, scored lower due to incomprehensive description and need to be improved further.

Moreover, the AGREE II Instrument did not provide a cut-off and lacked detailed information to distinguish the quality of CPGs (41). There were several types of cut-offs as following: 1) high quality (five to six domains scoring > 60%), average quality (three to four domains scoring > 60%) and low quality (two or less domains scoring > 60%) (14, 22, 40); 2) grade A (strongly recommended, six domains scoring > 60%), grade B (recommended, more than three domains scoring ranged from 30% ~ 60%) and grade C (not recommended, more than three domains scoring < 30%) (17); 3) when the AGREE II score ≥ 45, CPGs were included in the final extraction (42); 4) the recommendation levels (‘yes’, ‘yes with modifications’ and ‘no’) were given without additional division of CPGs’ quality (16); 5) ‘high quality’, ‘moderate quality’ and ‘low quality’ were divided by tertile according to mean of AGREE II & AGREE REX overall scores (37); 6) domains or overall scores < 50% indicated lower quality (43, 44); 7) overall score > 60% was classified as ‘recommended’, score between 30% ~ 60% as ‘recommended with modifications’, and score < 30% as ‘not recommended’ (38, 39). It might be needed that clear distinction and specified cut-off of quality level in the update of the AGREE II Instrument (41).

The update, external review and editorial independence serve as significant parts for CPGs. In this study, the references of included CPGs indicated that CPGs were not always based on the latest systematic reviews or primary studies. Robin et al. (45) found that most of the CPG methodological handbooks (62.9%) included updating intervals of two to five years. CPGs are time-sensitive for knowledge development and changes (37). Recommendations become outdated quickly, with 20% of recommendations turning outdated after three years (46). Several CPGs within this review (29, 31, 32, 34) were not updated over five years which may lead to a lag of information. As for the external review, three CPGs (24, 25, 34) were not published in a journal but by academic organizations on websites. Although journal publications underwent rigorous peer review, academic organizations and professional societies have procedures that were not always described in the CPGs. Moreover, some CPG authors did not disclosed their editorial independence within the CPGs. Sometimes the relevant information can be found on websites despite difficulty finding it. CPGs did not report as the framework of the standard formulation may be short of important information, weakening the transparency and reliability.

The CPGs held different views, which were possibly based on population differences. At first, for the nutrition assessment, there were differences across CPGs. In the ESPEN guideline (23), nutrition assessment, including muscle mass, nutritional intake, the degree of systemic inflammation, physical performance, and nutrition impact symptoms, was recommended for patients at risk of malnutrition by nutrition screening. But in the DGEM guideline (32) and the SEOM guideline (33), the assessment items were not that comprehensive. Secondly, there were discrepancies among CPGs in terms of protein intake. The protein intake should be above 1.2 g/kg/day in patients receiving RT with or without chemotherapy, according to the ASCO guideline (27), the UK guideline (31) and the COSA guideline (34); in the ESPEN guideline (23) and the CACA guideline (28), aim for protein intake was at least 1 g/kg/day and, if possible, up to 1.5 g/kg/day; the CSPEN guideline (30) suggested the target of protein intake for cancer patients was 1.0 to 2.0 g/kg/day; and the DGEM guideline (32) recommended that the protein/amino acid intake be 1.2-1.5 g/kg/day and the requirement may be higher (up to 2 g/kg/day) in the case of obvious inflammation. Therefore, our prudent advice is that protein intake should be above 1.2 g/kg/day and achieve1.5 g/kg/day if available. Thirdly, the DGEM guideline (32) and the SEOM guideline (33) considered that cancer patients had similar nutritional needs as the healthy population, about 25-30 kcal/kg/day. Still, in three CPGs (27, 31, 34), recommended energy intake should not be less than 125 kJ/kg/day (30 kcal/kg/day). Consequently, it is appropriate for HNC patients to intake 30 kcal/kg/day.

Given ambiguity in guidance and an absence of further verification, nutrients, pharmacological interventions, nutritional risk screening and nutrition assessment, emphasized the need for robust evidences. Most nutrients or drugs to improve nutritional status (e.g., appetite, taste, and reducing weight loss) for cancer patients were not recommended, due to lack of high-quality and large randomized controlled or cohort studies, similar to the finding of a previous study (38). Further studies are needed to explore whether and how the nutrients or drugs play a role in HNC patients’ malnutrition. Moreover, there is no uniformly used or specialized scale of nutritional risk screening and assessment for HNC patients. The AND guideline (26) recommended the Malnutrition Screening Tool and the Malnutrition Universal Screening Tool for nutritional risk screening; the DGEM guideline suggested the Nutritional risk screening 2002 and the Malnutrition Universal Screening Tool for use (32); and the COSA guideline (34) considered the Malnutrition Screening Tool as recommendation. Although the Nutritional Risk Screening 2002 (NRS-2002) scale has been widely used, the sensitivity for HNC patients receiving RT is still uncertain. The Patient-Generated Subjective Global Assessment (PG-SGA) and Subjective Global Assessment (SGA) were recommended in the reviewed CPGs (26, 34), but their applicability for HNC patients still needs to be verified.

## Limitations

There were several limitations to this study. Firstly, there were three appraisers to independently assess the CPGs while the AGREE II manual suggests that it be better if four appraisers are available. In the second place, the literature search was performed up to May 1, 2022, as the latest literature search was performed, there are no more new CPGs found. Thirdly, the AGREE II Instrument focuses on the comprehensiveness and intelligibility of the CPGs, not the quality of the recommendations or the clinical context (43). Besides, the overall assessment with the AGREE II Instrument is highly subjective (47). Last but not least, selection bias cannot be excluded in this study due to the language restrictions for Chinese, English, and German.

## Conclusion

In this study, the recommendations for nutrition of included oncology CPGs suggesting that nutrition is an essential component in peri-radiotherapy care. The quality of the included CPGs was highly heterogeneous. The discrepant recommendations from the whole process of nutrition management existed among CPGs. The CPG developers should adopt a multi-disciplinary approach, rely on evidence of high quality, and include the target population in the formulating recommendations. If the framework for CPG development and other supplementary materials cannot be reported within the main document, a trail or statement to its link should be listed. And beyond that, there are many kinds of evidence grading systems and recommendation strength methods, so we emphasize the need for comparing evaluation systems or developing a widely applicable system in the future. Using tools like the AGREE II Instrument to develop CPGs may be a good choice due to its scientific and unified criteria for quality reporting. At last, the development of CPGs should consider fundamental and medical resource allocation worldwide.

## Data availability statement

The original contributions presented in the study are included in the article/**Supplementary Material**. Further inquiries can be directed to the corresponding author.

## Author contributions

Conception and design, JZ, YK, and SY. Literature retrieval, literature screening, and data extraction, YK and XW. Guideline quality assessment, YK, XW, and GW. Data analysis and interpretation, JZ, YK, XW, YB, and JL. Manuscript writing, all authors. All authors contributed to the article and approved the submitted version.

## Acknowledgments

We thank Ms. Ruishuang Zheng and Mr. Fangyuan Zhang for their cogitative guidance and perceptive comments on this review.

## Conflict of interest

The authors declare that the research was conducted in the absence of any commercial or financial relationships that could be construed as a potential conflict of interest.

## Publisher’s note

All claims expressed in this article are solely those of the authors and do not necessarily represent those of their affiliated organizations, or those of the publisher, the editors and the reviewers. Any product that may be evaluated in this article, or claim that may be made by its manufacturer, is not guaranteed or endorsed by the publisher.
