# Supplementary material for: Nutrition management for patients with head and neck cancer during peri-radiotherapy: A systematic review and quality appraisal of clinical practice guidelines using the AGREE II instrument
Source: Front Oncol. 2022 Nov 29;12:974059. doi: 10.3389/fonc.2022.974059 (PMC9745777; doi:10.3389/fonc.2022.974059)
Supplement: Supplementary file 1 [file DataSheet_1.docx]

**Supplementary Materials 1: Search Strategies**

1. **PubMed Search Strategy**

Search Date: [March](javascript:;) 8, 2022

Final Search: #10

| Search | Query | No. of Results |
| --- | --- | --- |
| #1 | "Head and Neck Neoplasms"[MeSH Terms] | 337,249 |
| #2 | "head and neck neoplasm*"[All Fields] OR "cancer of head and neck"[All Fields] OR "cancer of the head and neck"[All Fields] OR "head and neck cancer*"[All Fields] OR "head and neck carcinoma*"[All Fields] OR "head and neck tumor*"[All Fields] OR "head and neck survivor*"[All Fields] | 76,336 |
| #3 | #1 OR #2 | 346,779 |
| #4 | nutrition*[All Fields] OR diet*[All Fields] OR malnutrition[All Fields] OR sarcopenia[All Fields] OR anorexia[All Fields] OR cachexia[All Fields] | 1,484,844 |
| #5 | "Radiotherapy"[MeSH Terms] | 201,601 |
| #6 | "Chemoradiotherapy"[MeSH Terms] | 18,630 |
| #7 | radiotherap*[All Fields] OR "radiation therap*"[All Fields] OR "radiation treatment*"[All Fields] OR chemoradiotherap*[All Fields] OR radiochemotherap*[All Fields] | 423,575 |
| #8 | #5 OR #6 OR #7 | 444,913 |
| #9 | guideline*[All Fields] OR CPG*[All Fields] OR "clinical practice guideline*"[All Fields] | 598,730 |
| #10 | #3 OR #4 OR #8 OR#9 | 114 |

1. **Cochrane Library Search Strategy**

Search Date: [March](javascript:;) 8, 2022

Final Search: #10

| Search | Query | No. of Results |
| --- | --- | --- |
| #1 | MeSH descriptor: [Head and Neck Neoplasms] explode all trees | 6,517 |
| #2 | ("head and neck neoplasm*"OR"cancer of head and neck"OR"cancer of the head and neck" OR "head and neck cancer*"OR"head and neckcarcinoma*"OR"head and neck tumor*" OR "head and neck survivor*") | 5,430 |
| #3 | #1 or #2 | 10,327 |
| #4 | (nutrition* OR diet* OR malnutrition OR sarcopenia OR anorexia OR cachexia) | 147,994 |
| #5 | MeSH descriptor: [Radiotherapy] explode all trees | 6,606 |
| #6 | MeSH descriptor: [Chemoradiotherapy] explode all trees | 1,149 |
| #7 | (radiotherap* OR chemoradiotherap* OR radiochemotherap* OR "radiation therap*" OR "radiation treatment*" | 39,437 |
| #8 | #5 OR #6 OR #7 | 39,746 |
| #9 | (guideline* OR CPG* OR "clinical practice guideline*") | 53,092 |
| #10 | #3 OR #4 OR #8 OR#9 | 26 |

1. **EMBASE Search Strategy**

Search Date: 2022

Final Search: #23

| Search | Query | No. of Results |
| --- | --- | --- |
| #1 | 'head and neck tumor'/exp | 366,265 |
| #2 | 'head and neck cancer'/exp | 202,735 |
| #3 | 'head and neck carcinoma'/exp | 84,879 |
| #4 | 'head and neck neoplasm*':ab,ti OR 'cancer of head and neck':ab,ti OR 'cancer of the head and neck':ab,ti OR 'head and neck cancer*':ab,ti OR 'head and neck carcinoma*':ab,ti OR 'head and neck tumor*':ab,ti OR 'head and neck survivor*':ab,ti | 49,573 |
| #5 | #1 OR #2 OR #3 OR #4 | 370,321 |
| #6 | 'nutrition'/exp | 2,549,722 |
| #7 | 'diet'/exp | 392,062 |
| #8 | 'sarcopenia'/exp | 15,935 |
| #9 | 'anorexia'/exp | 62,271 |
| #10 | 'cachexia'/exp | 16,543 |
| #11 | 'malnutrition'/exp | 191,790 |
| #12 | 'nutrition*':ab,ti OR 'diet*':ab,ti OR sarcopenia:ab,ti OR anorexia:ab,ti OR cachexia:ab,ti OR malnutrition:ab,ti | 1,203,142 |
| #13 | #6 OR #7 OR #8 OR #9 OR #10 OR #11 OR #12 | 3,089,724 |
| #14 | 'radiotherapy'/exp | 638,263 |
| #15 | 'chemoradiotherapy'/exp | 66,361 |
| #16 | radiotherap*:ab,ti OR chemoradiotherap*:ab,ti OR radiochemotherap*:ab,ti OR 'radiation therap*':ab,ti OR 'radiation treatment*':ab,ti | 428,804 |
| #17 | #14 OR #15 OR #16 | 748.860 |
| #18 | 'guideline'/exp | 142 |
| #19 | 'clinical practice guideline'/exp | 11 |
| #20 | 'cpg'/exp | 15 |
| #21 | guideline*:ab,ti OR cpg*:ab,ti OR 'clinical practice guideline*':ab,ti | 704,672 |
| #22 | #18 OR #19 OR #20 OR #21 | 704,715 |
| #23 | #5 AND #13 AND #17 AND #23 | 223 |

1. **Web of Science Search Strategy**

Search Date: [March](javascript:;) 8, 2022

Final Search: #5

| Search | Query | No. of Results |
| --- | --- | --- |
| #1 | TS=("head and neck cancer*" OR "head and neck neoplasm*" OR "head and neck tumor*" OR "head and neck carcinoma*" OR "head and neck cancer survivor*") | 93,277 |
| #2 | TS=(nutrition* OR diet* OR malnutrition OR sarcopenia OR anorexia OR cachexia) | 2,824,652 |
| #3 | TS=(radiotherap* OR chemoradiotherap* OR radiochemotherap* OR “radiation therap*” OR “radiation treatment*”) | 603,312 |
| #4 | TS=(guideline* OR CPG* OR “clinical practice guideline*”) | 895,399 |
| #5 | #1 OR #2 OR #3 OR#4 | 146 |

1. **SinoMed Search Strategy**

Search Date: 2022

Final Search: #16

| Search | Query | No. of Results |
| --- | --- | --- |
| #1 | "头颈部肿瘤"[不加权:扩展] | 550,025 |
| #2 | "头%瘤"[常用字段:智能] OR "头%癌%"[常用字段:智能] OR "头%癌"[常用字段:智能] OR "颈癌"[常用字段:智能] OR "颈肿瘤"[常用字段:智能] OR "头癌"[常用字段:智能] AND "头肿瘤"[常用字段:智能] | 261122 |
| #3 | #1 OR #2 | 261122 |
| #4 | "营养不良"[不加权:扩展] | 26532 |
| #5 | "恶病质"[不加权:扩展] | 1109 |
| #6 | "膳食"[不加权:扩展] | 12229 |
| #7 | "营养"[常用字段:智能] OR "饮食"[常用字段:智能] OR "膳食"[常用字段:智能] OR "营养不良"[常用字段:智能] OR "肌肉减少症"[常用字段:智能] OR "厌食症"[常用字段:智能] OR "恶液质"[常用字段:智能] OR "恶病质"[常用字段:智能] | 366367 |
| #8 | #4 OR #5 OR #6 OR #7 | 366367 |
| #9 | "化放疗"[不加权:扩展] | 2 |
| #10 | "放射疗法"[不加权:扩展] | 21982 |
| #11 | "放%疗"[常用字段:智能] OR "放%疗%"[常用字段:智能] OR "放疗"[常用字段:智能] OR "%放疗"[常用字段:智能] OR "%放化疗"[常用字段:智能] | 211170 |
| #12 | #9 OR #10 OR #11 | 214901 |
| #13 | "指南"[不加权:扩展] | 876 |
| #14 | "指南"[常用字段:智能] | 49570 |
| #15 | #13 OR #14 | 49570 |
| #16 | #3 AND #8 AND #12 AND #15 | 13 |

1. **CNKI Search Strategy**

Search Date: [March](javascript:;) 8, 2022

((((((((((TKA=营养 OR TKA=饮食) OR TKA=膳食) OR TKA=营养不良) OR TKA=恶液质) OR TKA=恶病质) OR TKA=肌肉减少症) OR TKA=厌食症) AND TKA=指南) AND ((TKA=头颈部肿瘤 OR TKA=头颈肿瘤) OR TKA=头颈癌)) AND ((((TKA=放疗 OR TKA=放射治疗) OR TKA=放射疗法) OR TKA=放化疗) OR TKA=化放疗))/62

1. **WanF****ang Search Strategy**

Search Date: [March](javascript:;) 8, 2022

主题: (指南) AND 主题: (放疗 OR 放射治疗 OR 放射疗法 OR 放化疗 OR 化放疗) AND 主题: ( 头颈部肿瘤 OR 头颈肿瘤 OR 头颈癌 ) AND 主题:(营养 OR 饮食 OR 膳食 OR 营养不良 OR 肌肉减少症 OR 恶液质 OR 恶病质 OR 厌食症)/17

1. **Vip Search Strategy**

Search Date: 2022

((((((((((U=营养 OR U=饮食) OR U=膳食) OR U=营养不良) OR U=恶液质) OR U=恶病质) OR U=肌肉减少症) OR U=厌食症) AND U=指南) AND ((U=头颈部肿瘤 OR U=头颈肿瘤) OR U=头颈癌)) AND ((((U=放疗 OR U=放射治疗) OR U=放射疗法) OR U=放化疗) OR U=化放疗))/9
